# Efficacy of recanalization therapy for ischemic stroke: multicenter hospital network experience

**DOI:** 10.1007/s11547-023-01599-5

**Published:** 2023-02-08

**Authors:** Francesco Briganti, Mario Tortora, Fabio Tortora, Andrea Elefante, Giovanni Loiudice, Mariano Marseglia, Giuseppe Buono, Michele Rizzuti, Rosa Iodice, Fiore Manganelli, Francesco Diurno, Fabio Giuliano Numis, Luigi Ferrara, Carlo Bruno, Alessandro Bresciani, Ferdinando Caranci, Donatella Franco, Carlo Vaiano, Gaetano D’Onofrio, Pasquale Scala, Rosa Raucci, Eufrasia Silvestro

**Affiliations:** 1 Dipartimento di Scienze Biomediche Avanzate, Università degli Studi di Napoli Federico II, Naples, Italy; 2 ASL Napoli2 Nord: Ospedali Pozzuoli, Frattamaggiore, Giugliano Italy; 3 Dipartimento di Medicina di Precisione, Università degli Studi della Campania “Luigi Vanvitelli”, Naples, Italy; 4 ASL Napoli3 Sud: Ospedale di Castellammare, Castellammare di Stabia, Italy; 5 Asl Caserta: Ospedale di Aversa, Aversa, Italy

**Keywords:** Stroke, Mechanical thrombectomy, Intravenous thrombolysis, HUB and SPOKE network

## Abstract

**Purpose:**

Stroke is a leading cause of long-term disability with high mortality rate in the first year after the event. In Campania, mechanical thrombectomy treatment significantly increases in the last 3 years, as well as hospitals delivering acute stroke treatments. The aim of this study is to demonstrate how a full opening of our stroke network improves stroke management and stroked patients’ survival in Campania.

**Material and methods:**

In Federico II University Hospital of Naples acting as a HUB center of 7 peripheral SPOKE hospitals in regional territory, 68 patients with acute ischemic stroke were evaluated with NIHSS and m-RS clinical scores and neuroradiological ASPECT scores, from January 1 to December 31, 2021. At hospital discharge, NIHSS score and three months after m-RS score were re-assessed to evaluate the therapeutic effects.

**Results:**

Forty-two of 68 patients (63%) admitted to our hub center had ischemic acute stroke at CT evaluation; 29 patients had ASPECT score > 7 (69%), and 6 a score < 7 (14%). At admission, NIHSS score mean value was 10.75, and m-RS score mean value was 0.74. At discharge, NIHSS score mean value was 7.09. After three months, m-RS score mean value was 0.74.

**Discussion:**

The inter-company agreement between Federico II University and several peripheral hospitals allows an absolute and relative increase in endovascular mechanical thrombectomy and intravenous thrombolysis procedures, with a relative prevalence of mechanical thrombectomy. A regional implementation of the stroke multi-disciplinary care system is hardly needed to ensure the optimum treatment for the largest number of patients, improving patient’s outcome.

## Introduction

Stroke is a significant cause of morbidity and mortality [1]; it is the second cause of death and the third cause of combined death-disability in Italy. The Italian Stroke Organization (formerly ISO, actually ISA-AII) and the Ministry of Health report an annual incidence of 90.000 cases. One month mortality rate has been estimated around 20–30%; about 40–50% of people die within the first year from the event. Stroke is a leading cause of serious long-term disability in 75% of patients with reduced mobility in more than half of stroke survivors aged 65 and older [2].

The Italian Ministry of Health approved intravenous thrombolysis (IVT) in 2003; mechanical thrombectomy (MT) was permitted from 2015 as recanalization strategy with or without IVT, when not contraindicated to treat ischemic stroke [3].

According to the latest Italian national reports [4], the Campania region was the Italian tail end. Considering about 1390 patients suitable for thrombolytic treatment, less than 10% were treated from January 1 to December 31, 2017; in the same period, of 694 patients eligible for endarterectomy treatment (EA), only 3.3% underwent surgical treatment. The Campania region had four centers able to perform intravenous thrombolysis treatment and only one center active for mechanical thrombectomy.

In the last three years, IVT treatments are increased (from 140 cases in 2017 to 378 in 2020), MTs increased dramatically 18-folds (from 23 cases in 2017 to 430 in 2020), the global number of treated patients fourfold (from 157 in 2017 to 628 in 2020) and the number of hospitals delivering acute stroke treatments increased at least to 6. Despite these results, Campania region brings up the rear.

We aim to demonstrate how a full opening of our stroke network improves stroke management in Campania and increases the numbers of survival patients and their quality of life.

## Material and methods

In January 2021, the Interventional Neuroradiology Unit of the Federico II University Hospital of Naples, in association with the staff of Neurology Clinic, signed an inter-company agreement for the diagnosis and treatment of ischemic stroke.

This integrated HUB & SPOKE network recognizes the Federico II Neuroradiology Unit as the HUB center involving the following SPOKE hospitals from several peripheral towns: Giugliano (Spoke1); Frattamaggiore (Spoke2); Pozzuoli (Spoke3); Castellammare di Stabia (Spoke4); Nola (Spoke5); Aversa (Spoke6); Battipaglia (Spoke7).

From January 1 to December 31, 2021, sixty-eight (68) patients with suspected acute vascular ischemic attack were evaluated. Of these, 42 were really suffering from ischemic stroke and are included in the global study evaluations.

At admission, each patient underwent NIHSS and m-RS scores neurological evaluation and, through CT scan, the neuroradiological ASPECT score assessment to determine the best diagnostic goal and therapeutic treatment. At hospital discharge, we re-calculated NIHSS score and three months after m-RS score to check the therapeutic effects.

## Results

Sixty-eight (68) patients are divided according to the source spoke as follows: 22 (32,4%) Spoke1; 18 (26,5%) Spoke3; 12 (17,6%) Spoke2; 7 (10,3%) Spoke6; 5 (7,3%) Spoke5; 3 (4,4%) Spoke4; 1 (1,5%) Spoke7. CT scan showed: 42 (63,0%) patients with ischemic acute strokes; 22 (32,0%) subarachnoid hemorrhage; 4 (6,0%) intra-parenchymal hemorrhage.

In this regard, 26 hemorrhagic patients, detected in the SPOKE units, were not sent to HUB center, while 42 ischemic patients were sent to the HUB, starting with intravenous therapy.

**Fig. 1:**
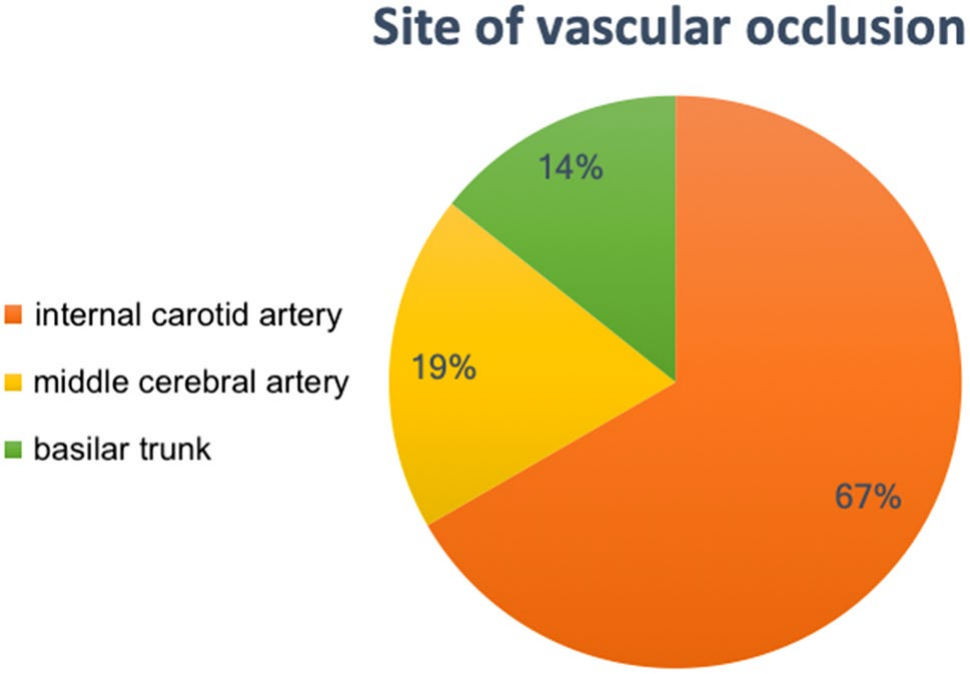
Pie chart illustrates the different occlusion sites in stroke cases treated at our institution

For each of 42 ischemic patients, the ASPECT score was calculated at admission and they were subdivided into two groups: the first one with a > ASPECT score (29; 69,0%) and second one with a < 7 ASPECT score (6; 14,0%). In seven out of 42 (17,0%) patients, ASPECT score was not evaluated (i.e., posterior circle stroke). The site of vascular occlusion is shown in Fig. 1.

**Fig. 2:**
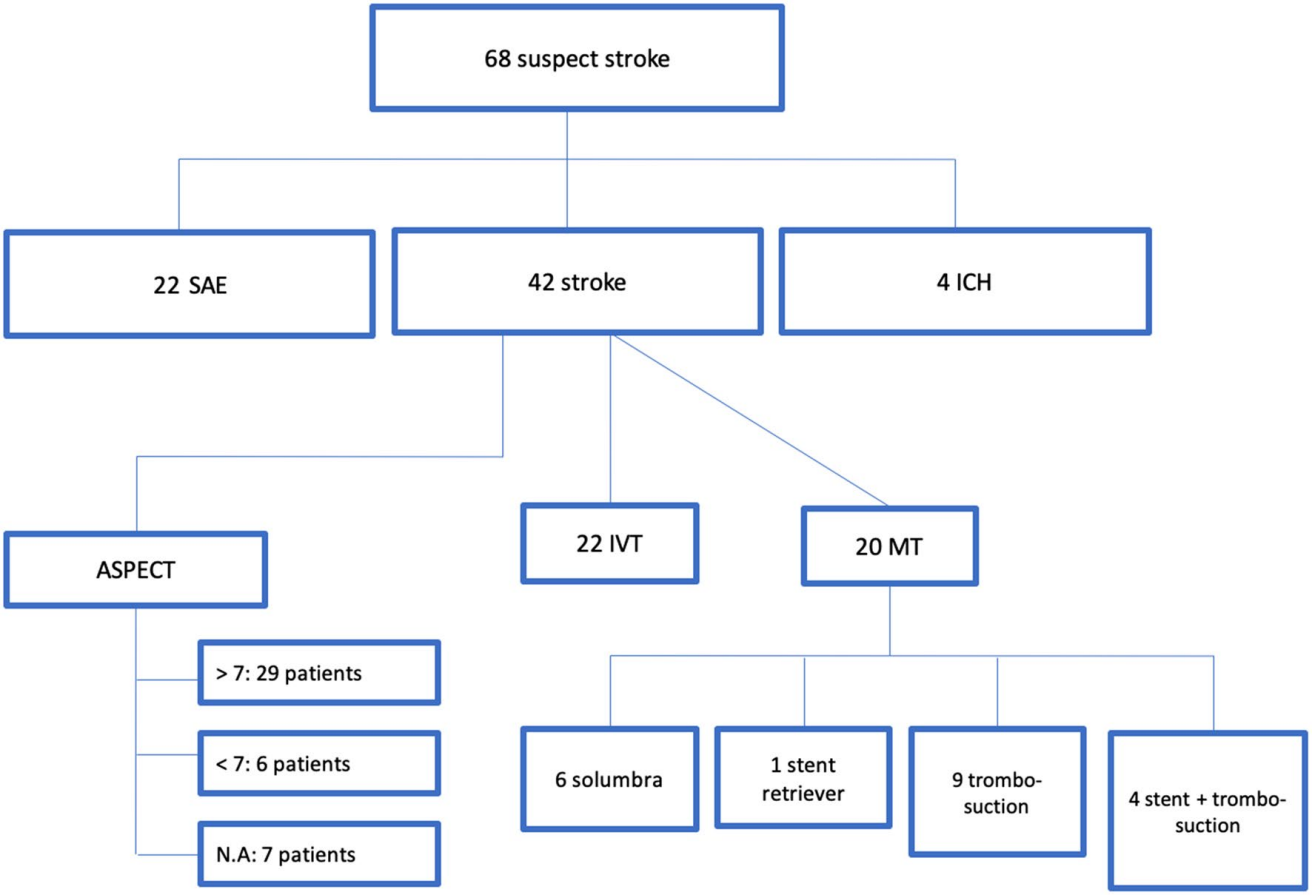
Diagram shows how suspected stroke cases with ASPECT evaluation and treatment modalities were handled

At admission, NIHSS score was 1–4 (minor stroke) in 14 (33,3%) patients; 5–15 (moderate) in 21 (50,0%) patients; 16–20 (moderate-severe) in 2 (4,8%) patients; > 21 (severe) in 5 (11,9%) patients. NIHSS mean value was 10.75 (min: 2; max: 29). At the same time, m-RS score mean value was 3.15 (min: 2; max: 6). Twenty (48,0%) patients underwent endovascular mechanical thrombectomy (MT). The following procedures were used: 6 (30%) SOLUMBRA technique; 1 (5%) stent retriever; 9 (45%) thrombosuction; 4 (20%) stenting and thrombosuction with different technique to SOLUMBRA (stent retriever in distal affixing to the thrombus and thrombus suction not in contact with the stent retriever). (Fig. 2) [9].

At discharge, NIHSS score was: 0 in 3 patients; 1–4 in 21 patients; 5–15 in 6 patients; 16–20 in 4 patients; > 21 in 2 patients. Six patients are dead. NIHSS mean value was 7.09 (min: 0; max: 21). At three months after attack, m-RS score mean value was 0.74 (min: 0; max: 3).

**Fig. 3:**
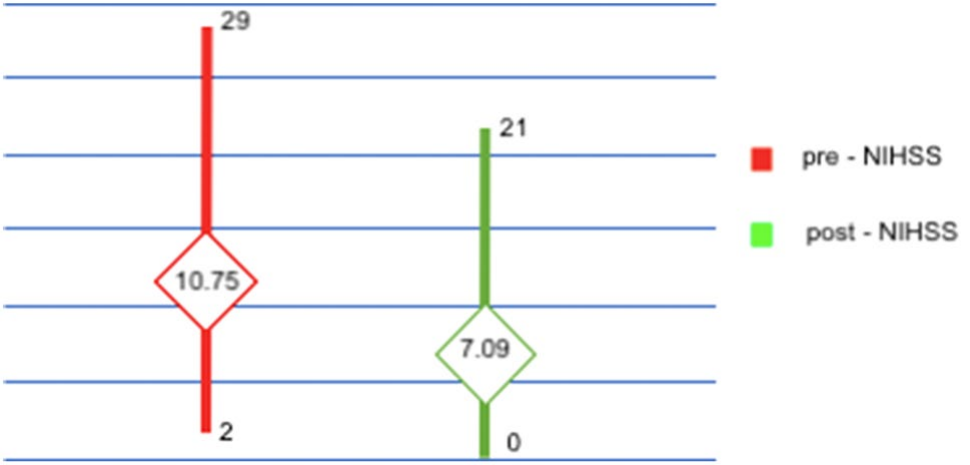
Ratings (mean value, maximum value and minimum value) NIHSS before and after treatment

**Fig. 4:**
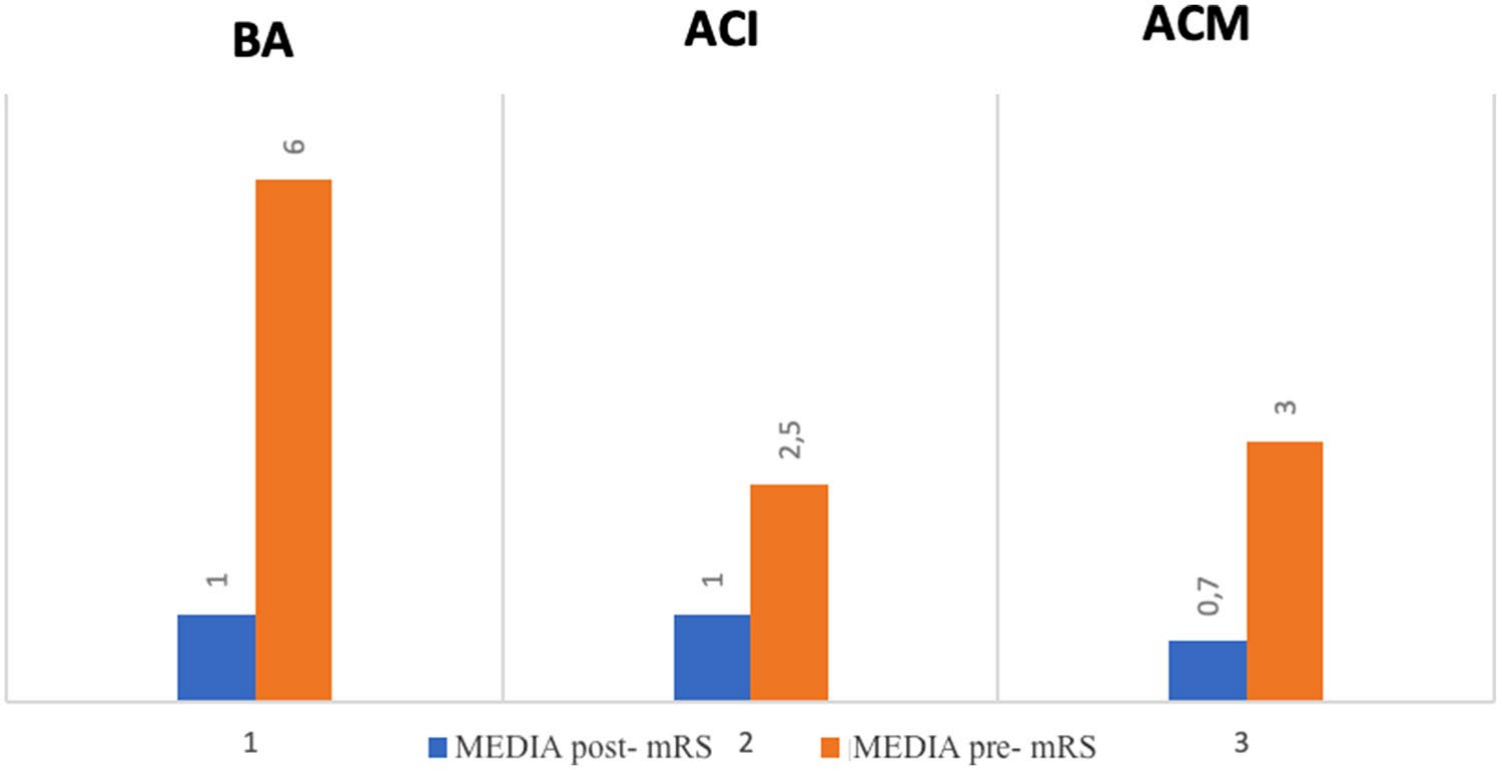
Ratings (mean value, maximum value and minimum value) mRS before and after treatment

The differences between pre- and post-procedural NIHSS and m-RS scores (the second one according to the site of vascular occlusion) are shown in Figs. 3 and 4.

## Discussion

From January 2021, in order to cope with the shortage of care units for acute cerebral ischemic pathology, the Interventional Neuroradiology Unit of the Federico II University of Naples signed an inter-company agreement with seven different hospitals diffused on regional territory. This integrated HUB & SPOKE network—that recognizes the Federico II Interventional Neuroradiology Unit as HUB center and territorial hospitals as SPOKEs—aims to connect the individual centers specialized in the management of ischemic stroke in order to guarantee an effective and rapid response to unmet need of population. Above all the most complex cases are managed at high-level and high-complexity centers (HUB) supported by the network of territorial services (SPOKES) operating on an outpatient basis. The HUB center provides a 24-h activity. According to these rules, for patients arriving at spoke units with suspected diagnosis of acute vascular ischemic stroke, the hub center was called. The Federico II Interventional Neuroradiology Unit (guaranteed h24; 7 days a week) activates the internal network consisting of the Neurological Clinic and anesthesiology support. For ischemic stroke, NIHSS and ASPECT scores were defined when it is possible. In case of hemorrhagic lesions in basal CT performed at SPOKE units and/or ASPECTS < 7, the patient will not be eligible for either intravenous or intra-arterial therapy. The patients detected as hemorrhagic stokes were not sent to HUB center. For patients who meet the criteria for IVT, without delaying intravenous therapy where indicated (recommendation I, A), we perform angio-CT (CTA) even in the absence of recent serum creatinine determination in patient without history of renal failure (recommendation IIa, B-NR).

So, we perform thrombolysis at 4.5 h after symptom onset after perfusion CT or DW/PW MRI detecting: hypoperfused tissue/ischemic core volume ratio > 1.2 (ECASS IV); ischemic core volume < 70 ml, tissue volume ratio > 1.2, absolute volume difference between hypoperfused tissue and ischemic core > 10 ml (EXTEND); PW/DW volume ratio > 1.2 and PW-DW volume ≥ 10 ml (EPITHET) [5–7].

We adopt the DEFUSE3 and DAWN criteria [8, 9] for the selection of patients for interventional procedure in case of more than 6 h onset of the symptoms. Specifically, according to the DEFUSE-3 study (< 16 h onset, < 90 year patients), we perform DWI/PWI sequences or perfusion CT scan on patients with the following selection criteria: infarct core < 70 ml, penumbra area > 15 ml, volume ratio of hypoperfusion area to infarct area > 1.8. According to the DAWN study (< 24 h onset), we evaluate mainly the infarct core, with clinical imaging mismatch (CIM) defined as one of the following on MR-DWI or CTP-rCBF maps:

(a) > 80 y/o, NIHSS > 10 + core < 21 mL;

(b) < 80 y/o, NIHSS > 10 + core < 31 mL;

(c) < 80 y/o, NIHSS > 20 + core < 51 mL.

In light of these guidelines and studies, we were able to treat a total of 20 patients with endovascular mechanical thrombectomy. In addition, we treated a basilar artery occlusion more than 12 hours (14) after the onset of symptoms with a very good result. Similarly, it was possible for us to treat a TANDEM case of carotid artery dissection with middle cerebral artery occlusion by having the possibility to treat first the dissective problem and then the occlusive one without vital delays in saving the brain parenchyma [10, 11].

Our data show, according to very fast growth trend in recanalization therapy in Campania Region, the main role of our stroke unit in order to ensure an adequate regional level of care. Of course, the efforts to be devoted to the stroke network in Campania are still hard. In fact, the ACT 70/2015 of the Italian government provides that a first-level SU should serve an area of ​​150,000–300,000 inhabitants, with a second-level SU for every 600,000–1,200,000 inhabitants [12]. In addition, the need for dedicated SU beds is calculated as approximately 1 bed for every 19,000 inhabitants. The Campania government received Law 70/2015 with LR 63/2019 [13] but the number of patients not hospitalized in the Stroke Unit remains dramatically high and far from the goal of 90% hospitalizations in the Stroke Unit, as established in the Plan Action for Stroke in Europe 2018–2030 [14].

Another crucial consideration comes from the critical analysis of IVT and MT trends. Both treatments had an absolute and relative increase over time; however, this increase was significantly higher for MT than for IVT, and surprisingly, in 2020, the relative rate of MT exceeded that of IVT, despite occlusions of blood vessels, large sizes account for only 24% to 38% of ischemic stroke.

This can have several explanations including the lack of a top-level stroke unit which limits the spread of thrombolysis to peripheral areas, in a drip-and-ship pattern [15]. Also in this regard, the identification of our stroke unit as a first-level HUB becomes fundamental. Furthermore, it is essential to rationalize the pre-hospital care system and adequately train new young neuroradiologists, qualified in stroke care starting from the Schools of Radiology and with subsequent masters in neuroradiological intervention. Keeping in mind the Regional Law n.249 of December 28, 2020, there is a strong shortening of neuroradiologists in our Region and, even more specifically of interventional neuroradiologists, specialized in the treatment of stroke [16].

In conclusion, stroke treatment in Campania is improving also thanks to the efforts and self-denial of the health workers of our stroke unit as well as those, still insufficient, already present in the area.

Moreover, it is well established that multi-disciplinary teams organized in stroke units are essential to obtain improved outcomes in comparison with conventional care in terms of long-term decrease in death, dependency and need for institutional care.

Therefore, a regional implementation of the stroke care system is hardly needed, in order to ensure treatment in the shortest time and to reduce the heavy personal, family, social and economic consequences of stroke for the largest number of patients.
